# Mortality in geese from organic and conventional production systems transported for slaughter in Germany: The relevance of distance and thermal stress

**DOI:** 10.1016/j.psj.2026.107701

**Published:** 2026-08-24

**Authors:** Benedikt Sauer, Isabelle Ruhnke, Katharina Charlotte Jensen, Kenan Hizal, Eva-Maria Saliu

**Affiliations:** a HNEE – Eberswalde University for Sustainable Development, Department for Landscape, Society and Economy, Animal Husbandry in Organic Farming, Eberswalde, Germany; b Faculty of Veterinary Medicine, Livestock Clinic - Division of Poultry, Freie Universität Berlin, Berlin, Germany; c Faculty of Veterinary Medicine, Institute of Veterinary Epidemiology and Biostatistics, Freie Universität Berlin, Berlin, Germany

**Keywords:** Abattoir, Animal welfare, DOA, Environment, Stress

## Abstract

While live poultry transport remains a major sector of animal transportation within the European Union, transport-related welfare in geese has received comparatively little scientific attention. Germany was the third largest goose producer in the EU, but the continuing decline in poultry slaughterhouse numbers increases transport distances. The aim of this study was to investigate the relevance of transport distance and thermal conditions on Dead-on-Arrival rates (DOA) in slaughter geese from organic and conventional production systems transported in Germany.

Data from 2009 to 2025 including 1,789 commercial transports of slaughter geese were retrospectively analysed, comprising more than 3.2 million animals. The median DOA rate across all analysed transports was 0.048 % (IQR: 0.00 % – 0.137 %). Transport distance ranged from 9.7 to 554.0 km and was not significantly associated with DOA rates. In a multivariable linear regression model with cluster-robust standard errors accounting for repeated transports from the same fattening farm, daily mean temperature was significantly positively associated with DOA rate (β = 0.0115 percentage points per 1°C increase, robust SE = 0.0031, p < 0.001), whereas transport distance, housing system, and year were not significantly associated with DOA rate. Separate analyses of enthalpy categories showed increasing median DOA rates with increasing enthalpy, with comparatively low rates at ≤50 kJ/kg (median = 0.047 %) and the highest rates at >58 kJ/kg (median = 0.28 %). These results demonstrate generally low DOA rates among slaughter geese in Germany while highlighting the need for real-time thermal monitoring and improved thermal risk management during transport to reduce mortality and improve animal welfare.

## Introduction

Global geese production was estimated to affect more than 803 million slaughtered animals in 2024, representing an increase of more than 62 % compared to 2004, with China accounting for the vast majority with approximately of 769 million geese per year (FAO, 2026). Official data from 2018 indicate that Germany ranked as the third-largest producer of geese in Europe, following Poland and Hungary, and ahead of France and the Czech Republic (FAO, 2026). Although geese play a comparatively minor role in the German poultry industry, a total of 452,200 geese were commercially slaughtered in Germany in 2024 (Statistisches Bundesamt, 2025b). The small scale of production and the resulting limited number of waterfowl-specialised slaughterhouses necessitate relatively long transport distances of live geese under varying conditions.

Longer transport distances may pose challenges for animal welfare and production logistics, particularly when suitable regional slaughter facilities are limited (Schaack et al., 2018; Bayer et al., 2023). This may be of relevance for goose production, where the limited availability of specialised slaughter facilities can substantially influence transport distances and durations.

Between 2010 and 2024, Germany’s poultry slaughter sector experienced marked structural shifts: medium-sized facilities (50 - 249 employees) declined by 52.9 - 57.1 %, while small abattoirs (20 - 49 employees) expanded from 8 to 18 (125 %). Concurrently, large-scale operations (500+ employees) surged by 200 – 400 % (Statistisches Bundesamt, 2026a), reflecting the EU-wide trend of decreasing numbers of slaughterhouses favouring larger ones (EFA, 2019; Massot et. al., 2021). The total number of registered poultry slaughterhouses decreased in Germany from 204 to 164 between 2010 and 2024. Of these, 91 of them were registered for goose slaughter, while per capita consumption of goose meat remained relatively constant at 0.2 – 0.3 kg per capita per year (Statistisches Bundesamt, 2025a; Statistisches Bundesamt, 2025b). Consequently, poultry intended for slaughter in Germany must be transported over longer distances to reach the nearest slaughterhouse (Redantz et al., 2023). It is well described in the literature that longer transport times are associated with increased mortality rates in poultry (Nijdam et al., 2004; Voslářová et al., 2007b; Mitchell and Kettlewell, 2009; Cockram and Dulal, 2018).

To measure mortality during transport to slaughter, the indicator “Dead-on-Arrival” (DOA) is commonly used. DOA indicates how many animals died during transport, known as transport deaths, and is considered the most relevant “iceberg indicator” for poultry welfare during transportation (EFSA, 2022). This indicator intends to reflect several animal welfare issues at once and provides a quick assessment of overall animal welfare, like the tip of an iceberg points to a larger, hidden problem. The DOA rate is recorded in most European countries by official veterinarians and is therefore considered a reliable indicator to compare transportation circumstances between countries and for taking measures to protect animal welfare (Valkova, 2022).

A study published in 2024 on broiler chicken transport within Germany found no correlation between transport distance (4 - 450 km) and the DOA rate (Gickel et al., 2024). The researchers concluded that the overall low mean DOA rate (0.09 %) in German broiler transports might indicate high standards of transport practices in Germany, hence factors such as environmental temperature on truck, flock behaviour during rearing (active or calm), loading procedures, and lairage time are adequate and provide suitable conditions to maintain a low DOA rate in German broilers, although these variables were not assessed (Gickel et al., 2024). Similarly, the DOA for turkeys transported to slaughter in the first half of 2022 was 0.122 % in Germany (Gickel et al., 2025). German farmers who rear laying hens also struggle with the slaughterhouse infrastructure, as only 63 slaughterhouses for laying hens are available in the country (Redantz et al., 2023). While research on welfare-relevant transportation-related factors in broilers, layers and turkeys has been conducted in many countries, insights for waterfowl are scarce: Two studies were carried out in the Czech Republic between 1997 and 2006 (Voslářová et al., 2007a), and between 2010 and 2019 (Valkova, 2022), involving a total of 76,811 and 37,691 geese respectively, transported for slaughter. The latter study reported a comparatively low DOA rate in geese of 0.003 %, particularly when compared to other poultry categories, such as layers, which exhibited the highest mortality rate of 0.507 % in the same area and time frame (Valkova, 2022). Furthermore, a significant increase in the DOA rate among these geese was observed for transport distances exceeding 300 km (0.253 %) compared to distances up to 300 km (0.021 – 0.053 %), indicating that reducing transport distance can substantially decrease transport-related losses (Voslářová et al., 2007a). Studies from Czechia and Egypt on duck transport reported DOA rates of 0.052 % (≤ 50 km) to 0.105 % (> 50 km) (Voslářová et al., 2016), 0.133 % (≤ 50 km) to 0.325 % (> 50 km) (Justova et al., 2025), and 3.33 % (≤ 50 km) to 6.66 % (> 50 km) (Mousa-Balabel and Mohamed, 2011). Across all studies**,** DOA rates doubled to sextupled when transport distances exceeded 50 km, confirming distance as a critical risk factor for waterfowl. The most recent study was based on datasets of slaughter-ducks (n = 28,012,184) being transported for ≤ 50 – 300 km lacking detailed information on body weight, stocking density in transport crates, and individual vehicle configurations, although all vehicles complied with Regulation (EC) No 1/2005. During the monitored period, ambient temperatures ranged from 0.5 - 18.6°C (Justova et al., 2025). In contrast, the Egyptian study was conducted under different legislative requirements regarding fitness for transport, as well as under distinct environmental conditions. Ducks (n = 210) were transported at outside temperatures of 21 ± 4°C and inside crate temperatures of 26 ± 2°C, with a relative humidity of 75 %, and a stocking density of ten birds per crate (1.3 × 0.7 × 0.25 m) and an average body weight of 2.3 kg (Mousa-Balabel and Mohamed, 2011).

In addition to specified transportation standards and legal regulations, the ambient temperature is one of the key factors influencing the DOA rate. In accordance with Regulation (EC) 1/2005, poultry may only be transported at temperatures between 5 and 30°C, with a measurement tolerance of ±5°C (Council of the European Union, 2005). High temperatures in combination with long transport times in particular lead to increased thermal stress and thus to a higher risk of transport-related losses (Nijdam et al., 2004). Heat stress in poultry can be caused not only by high ambient temperatures, but also by high humidity, low air movement, or solar radiation (Oladokun and Adewole, 2022). The specific enthalpy of moist air can be used as a qualitative indicator to assess the thermal environment of poultry and can be calculated using the Albright Formula (Rodrigues et al., 2011). The higher the temperature and relative humidity, the faster critical enthalpy values are reached. Values above 67 kJ/kg (70 % humidity, 27°C) are known to cause severe heat stress in poultry (DWD, 2022). Severe heat stress describes a critical state in which the bird’s physiological capacity to regulate body temperature is markedly impaired, representing a “danger zone” (EFSA, 2022). As a result, birds may no longer be able to dissipate excess body heat, leading to a rise in internal body temperature. This is accompanied by a marked increase in respiratory rate to enhance evaporative cooling, which may become insufficient under high humidity and can result in physiological disturbances such as respiratory alkalosis (Pontara Vilas Boas Ribeiro et al., 2020). Accordingly, studies on the transport of poultry have reported seasonal differences in mortality rates. In ducks, mortality increased significantly from 0.072 % in winter to 0.090 % in summer (Voslářová et al., 2016). Similarly, in turkeys, higher DOA rates were observed in summer (maximum 1.48 %) compared to winter (maximum 1.34 %) (Gickel et al., 2025).

Overall, the relation between DOA rate, transport distance and thermal stress in geese is understudied globally and to our best knowledge there is no data published from Germany, despite the country’s relevance as a major European goose meat producer, the high animal welfare standards, and climatic conditions that are representative of Central Europe. In Germany, geese are typically slaughtered at mid-fattening around 16 weeks of age, which represents the standard production stage compared to earlier (8 – 10 weeks) or later fattening periods (28 – 32 weeks) (Golze, 2005; Hirt et al., 2023). Adult body weight generally ranges between 5.0 and 6.5 kg and is influenced by factors such as genetic background, feeding regime, sex, age, and management conditions. Geese exhibit rapid early growth, reaching approximately 70 – 80 % of their mature body weight by 8 – 9 weeks of age, compared to around 40 % in turkeys at the same age (Eroğlu and Erişir, 2022). These distinct growth patterns and bodyweights indicate species-specific physiological conditions, limiting direct extrapolation from other poultry species, particularly turkeys. This represents a critical knowledge gap for an often-overlooked poultry species. Therefore, the aim of this study was to investigate impact of transport distance, environmental temperature and enthalpy on the transport of slaughter geese in Germany.

## Materials and methods

### *Study data*

Retrospective evaluation of transportation data obtained by a German waterfowl slaughterhouse, based on official records by German Veterinarian Authorities from the years 2009 to 2025, was used as dataset for this study. These records were documented daily by official veterinary personnel after each transport at the slaughterhouse. For each transport, the dataset included the place of origin, housing system (conventional or organic), the total number of geese loaded and the number of geese that died during transport. Retrospectively, the official data was amended for the purpose of this study by the following parameters: transport distance of the flock from the place of load to the slaughterhouse (km), temperature (°C) and enthalpy (kJ/kg) on the day of transport, as well as the DOA rate (%) with details as follows:

The DOA rate (%) was calculated for each transport shipment by dividing the number of geese that died during transport by the total number of transported geese per shipment and multiplying the result by 100 (KTBL, 2020d). The transport distance for each transport was calculated using a route planner for a 40-ton truck (https://reiseplanung.de). This route-planning system is recommended by the State Working Group for Consumer Protection (Länderarbeitsgemeinschaft Verbraucherschutz) to German official veterinarians for verifying transport distances (LAV, 2024). It therefore describes the likely, not the actual transport duration. Hence, individual deviations during the transport process, such as traffic jams or extended waiting times, could not be considered. Furthermore, potential variation in animal loading, driving behaviour of the personnel, truck type, stocking density of the transport cages and individual lairage times for the animals at the abattoir could not be included in the analysis, due to lack of data. According to instructions from the veterinary staff, lairage times should be kept as short as possible so that, in accordance with legal requirements, the poultry is slaughtered within two hours of arrival; otherwise, the poultry must be provided with water (TierSchlV, 2012). For most shipments (n = 1559), the average carcass weight of the geese was recorded – this averages 4.43 kg (SD ± 0.55). With a slaughter yield of 73–74 %, the animals had a live weight of approximately 6 kg prior to slaughter – a weight reached at an average fattening period of 16 – 17 weeks (DLG, 2021). This is consistent with the statements made by the slaughterhouse operator, who noted that the animals were generally slaughtered at mid-fattening. The animals were caught and loaded in the (late) evening hours of the previous day to be transported overnight to the abattoir; slaughter began daily at around 06.30 am.

The data on the average daily mean temperature (T) and relative humidity (RH) were used from the rdwd package in R (Boessenkool, 2025). This package provides historical climate data from the German Weather Service (Deutscher Wetterdienst, DWD). The date of each transport was used to determine these parameters from a weather station within a radius of 10 - 15 km to the slaughterhouse. The reported values were based on daily averages and may therefore not precisely represent the environmental conditions within the truck at the specific time of transport, nor the actual climatic conditions encountered along the transport routes. Specific air enthalpy (h, kJ/kg dry air) was calculated using the Albright Formula (Rodrigues et al., 2011) as

h=1.006×T+w×(2501+1.805×T)

where w denotes humidity ratio (kg water per kg dry air) and T denotes temperature (°C). The humidity ratio was derived from RH and t using the Magnus equation $e_{s}=\;6.112\;\cdot \exp\frac{\left(17.62\;\cdot \;T\right)}{\left(243.12\;+\;T\right)}$, followed by the calculation of actual vapour pressure. This calculation was performed in R (version 4.4.1; R Core Team, 2024), implemented via RStudio (version 2025.09.1 + 401; Posit Software, Boston, MA, USA). Only complete datasets were considered in the analysis. Due to missing humidity data**,** 6 transports were excluded from the enthalpy-specific evaluation. For all other analyses, the full dataset comprising 1,789 transports (3,237,022 geese) was used.

### *Statistics*

Calculations were performed using IBM SPSS Statistics Version 31.0.1.0 (IBM Corp., 2024) and R (version 4.4.1; (R Core Team, 2024)) using the R Studio interface. After data cleaning was performed by checking for missing values and outliers, variables were analysed descriptively. Normality was tested using the Shapiro-Wilk test. The outcome variable, DOA, showed a zero-inflated distribution. The percentages of transports that were in line with established thresholds were displayed using bar charts (Figure 1). In the absence of goose-specific thresholds, the following values were used as comporative benchmarks: DOA target and alarm values for turkeys, laying hens and broilers of the Board of Trustees for Technology and Construction in Agriculture (Kuratorium für Technik und Bauwesen in der Landwirtschaft) (KTBL, 2020a; KTBL, 2020b; KTBL, 2020c), the alarm value for broilers defined by the German Working Group of the Federal States on Consumer Protection (LAV, 2025) and the target value from the European Food Safety Authority for all domestic birds (EFSA, 2022). This approach follows Gickel et al., who applied it to broiler chickens and turkeys, and is extended here to geese to contextualize the present results (Gickel et al., 2024; Gickel et al., 2025). The association between the DOA rate and potential influencing factors was analysed using a multivariable linear regression model with daily mean temperature, transport distance, housing system (conventional vs. organic), and year as explanatory variables. Because the dataset contained repeated transports from the same fattening farm, cluster-robust (sandwich) standard errors clustered at farm level were used to account for the non-independence of observations within farms; this approach relaxes the distributional assumptions of ordinary least squares and is robust to the right-skewed, heteroscedastic nature of the DOA rate. Multicollinearity among predictors was assessed using variance inflation factors, all of which were below 1.2, indicating no relevant collinearity. Year was modelled as a continuous term to test for a linear temporal trend. Linear regression analyses were performed in R, using the sandwich (Zeileis, 2004) and lmtest packages (Zeileis and Hothorn, 2002) for robust inference.

Additionally, as daily mean temperature and enthalpy were correlated and could not be included in the same model, the distribution of DOA across enthalpy categories was assessed using the Kruskal-Wallis test followed by posthoc pairwise comparisons with Bonferroni-adjusted p-values. Statistical significance was set to p < 0.05.

## Results

A total of 1,789 slaughter goose transports were evaluated, with an average of 1,809.4 (SD ± 570.1) geese per transport. Each transport involved a single loading event at one fattening farm, with no intermediate stops en route to the slaughterhouse using an approved transport operator in accordance with Regulation (EC) No 1/2005. Due to seasonal demand for goose meat in Germany, transport data were obtained during the second half of each calendar year (July-December).

***1. Description of DOA***

The DOA rate across all analysed slaughter geese transports (n = 1,789) had a median of 0.048 % (IQR: 0.00 % – 0.137 %), with a mean of 0.161 % (SD ± 0.616 %), reflecting the presence of outliers (n = 18) with comparatively high DOA rates (> 2 %). Notably, 41.5 % of all transports recorded a DOA rate of 0.0 %, indicating no mortality during transport. The median value (0.048 %) was below the KTBL target value of 0.05 % (KTBL, 2020a; KTBL, 2020b; KTBL, 2020c), with 52.4 % of transports falling within this threshold. In total, 68.2 % of transports remained below the limit of 0.1 % (EFSA, 2022), while 95.1 % were below the alarm threshold of 0.55 % (Fig. 1).

***2. Associations of transport distance, daily mean temperature, production system, and year with DOA***

**Figure 1:**
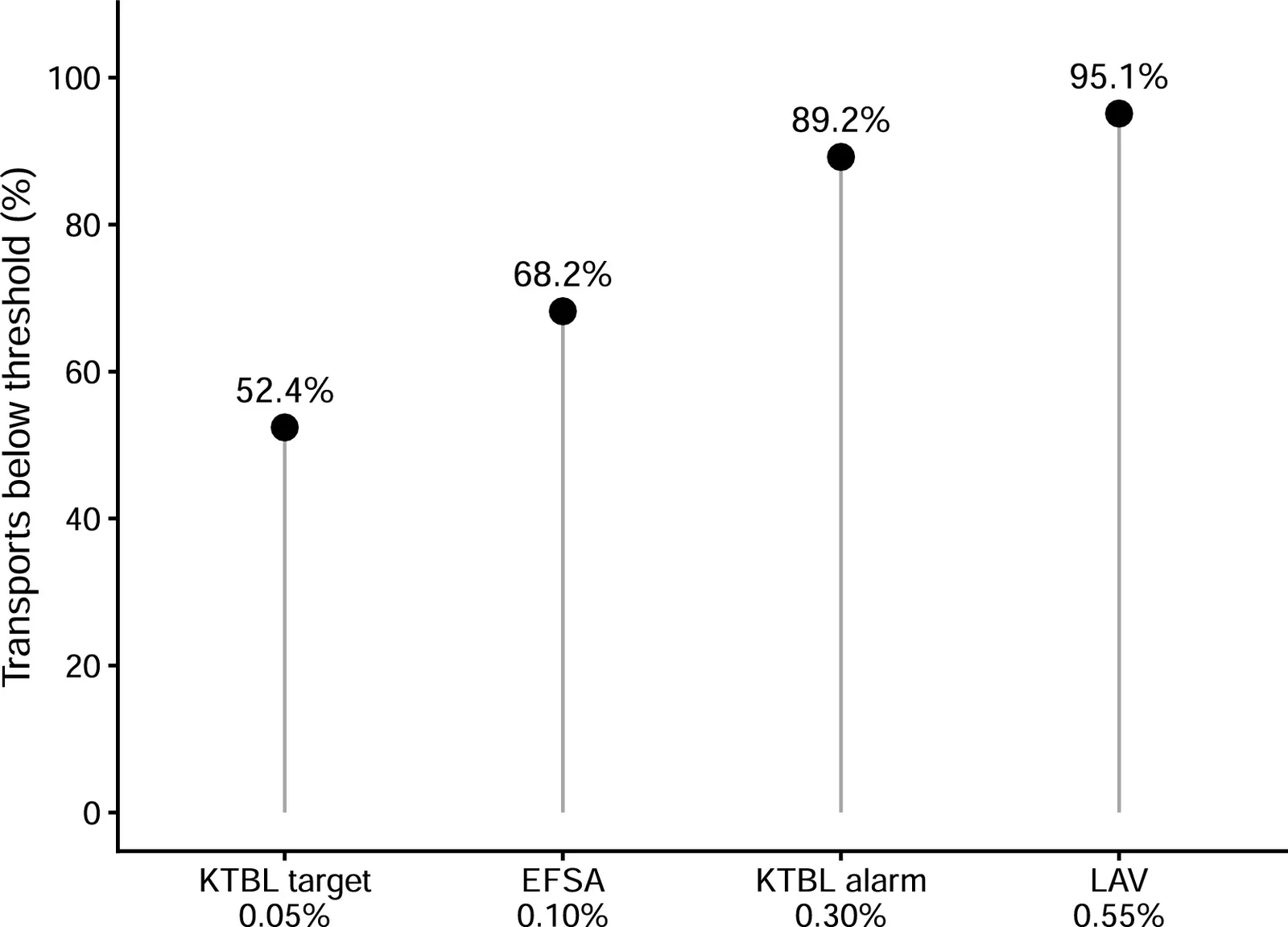
Percentage of slaughter goose transports (n = 1,789) in the dataset with DOA rates at or below published Dead-on-Arrival (DOA) benchmark values of the European Food Safety Authority (EFSA, 2022), the Board of Trustees for Technology and Construction in Agriculture (Kuratorium für Technik und Bauwesen in der Landwirtschaft*,*KTBL, 2020a–d), and the German Working Group of the Federal States on Consumer Protection (Länderarbeitsgemeinschaft Verbraucherschutz, LAV, 2025).

Transport distances between the fattening farms and the slaughterhouse ranged from 9.7 to 554.0 km, with a median of 285.5 km (IQR: 149.5–378.7 km). Daily mean temperature during the observation period ranged from −13.2 to 28.3°C, with an overall mean of 12.1 ± 7.5°C.

Median DOA was 0.049 % (IQR: 0.000–0.140 %; range: 0.000–15.000 %) in conventionally raised flocks and 0.042 % (IQR: 0.000–0.130 %; range: 0.000–2.110 %) in organically raised flocks.

In the cluster-robust regression model, higher daily mean ambient temperature was associated with higher DOA (Table 1). While the linear model indicated a significant positive association between daily mean temperature and DOA rate across the observed range, the relationship was not uniform: as illustrated in Figure 2, DOA rates remained consistently low across the low and intermediate temperature ranges and increased mainly at high daily mean temperatures (above approximately 18°C). The model showed no significant associations between DOA and transport distance, production system, or year (Table 1). Cluster-robust standard errors accounted for repeated transport observations from the same fattening farm.

***3 DOA across environmental enthalpy categories***

**Table 1:** Cluster-robust regression model for factors associated with Dead-on-Arrival (DOA)^2^ rate.

Predictor	Estimate^1^	Robust SE	*P*-value
Intercept	0.8099	6.7749	0.905
Daily mean temperature (°C)	0.0115	0.0031	< 0.001
Transport distance (km)	−0.00018	0.00016	0.260
Production system organic^3^	−0.0452	0.0470	0.337
Transportation year (2009-2025)	−0.00037	0.00335	0.913

1 Estimates (β), robust standard errors (SE), and P-values are shown. Cluster-robust SE were calculated at the fattening-farm level to account for repeated transport observations from the same farm.

2 DOA was entered into the model as a percentage; therefore, β estimates represent changes in percentage points.

3 Conventional production was the reference category.

**Figure 2:**
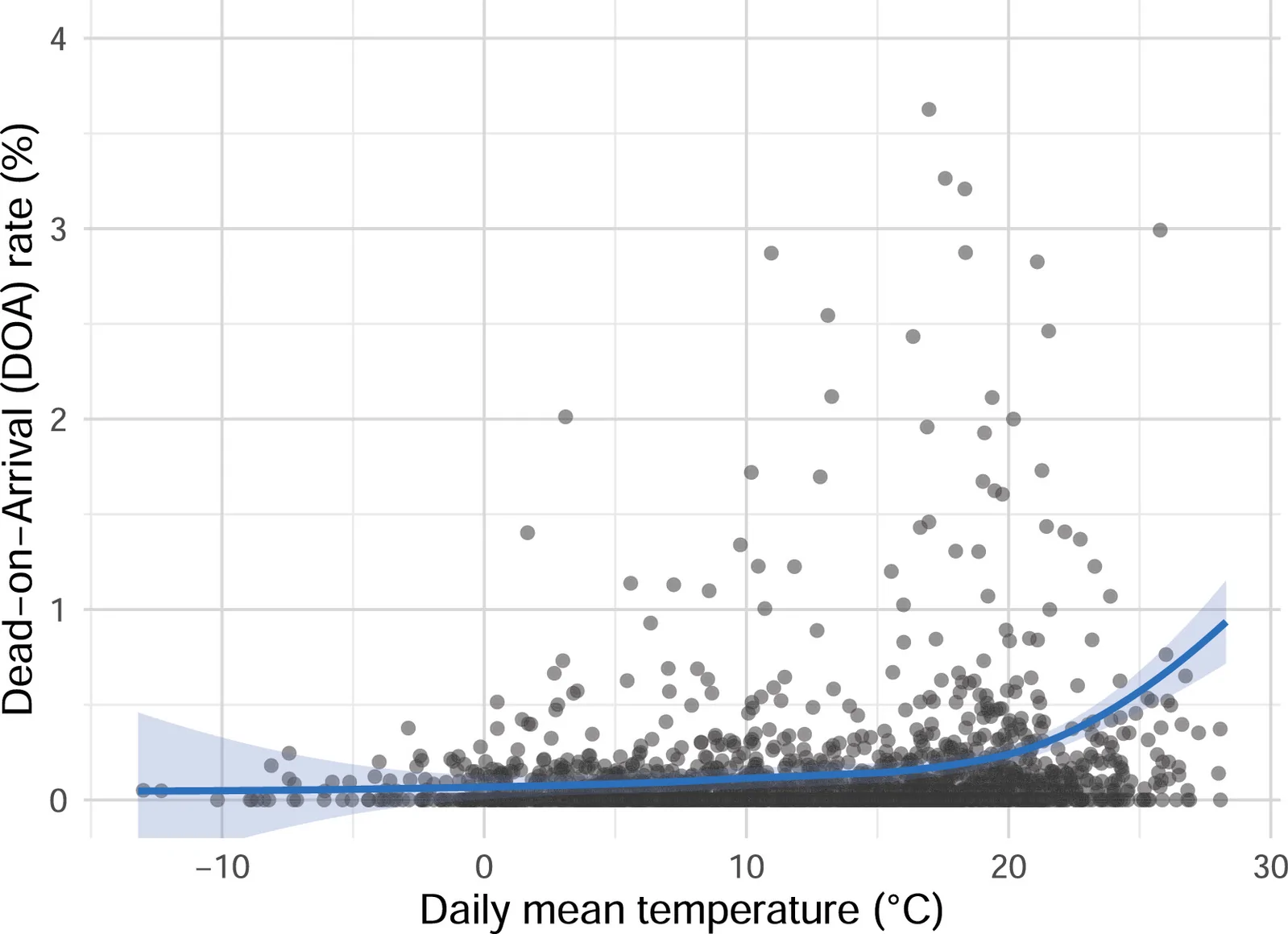
Association between daily mean temperature and Dead-on-Arrival (DOA) rate in slaughter geese transports. Grey points represent individual transport events (n = 1,789). The blue line represents a locally estimated scatterplot smoothing (LOESS) curve, and the shaded area indicates the corresponding 95 % confidence interval.

*A*s an additional analysis complementing the primary regression model, DOA was compared across three environmental enthalpy categories (≤50, >50 to ≤58, and >58 kJ/kg; Fig. 3). Of the 1,783 transport events, 1,652 (92.7 %) occurred at enthalpy values ≤50 kJ/kg, 119 (6.7 %) at values >50 to ≤58 kJ/kg, and 12 (0.7 %) at values >58 kJ/kg. Median DOA increased across the three categories, from 0.047 % (IQR: 0.000–0.127 %) at ≤50 kJ/kg to 0.130 % (IQR: 0.047–0.314 %) at >50 to ≤58 kJ/kg and 0.281 % (IQR: 0.044–0.553 %) at >58 kJ/kg.

**Figure 3:**
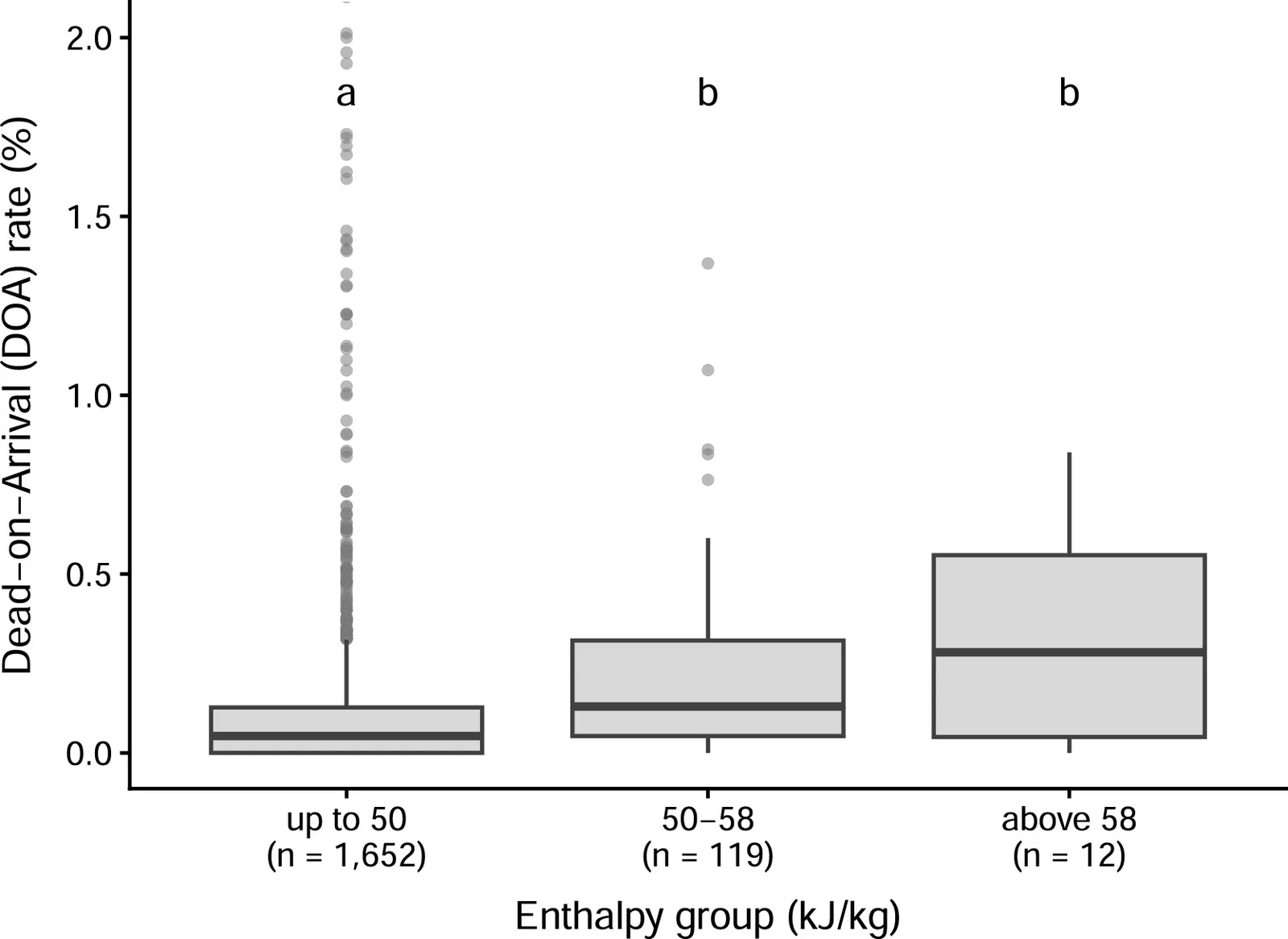
Dead-on-Arrival (DOA) rates across three enthalpy groups. Boxes represent the interquartile range, horizontal lines indicate the median, and whiskers extend to 1.5 times the interquartile range. Different letters indicate significant differences between groups based on Bonferroni-adjusted pairwise comparisons following the Kruskal–Wallis test (p < 0.05). Sample sizes are provided for each enthalpy group.

The distribution of DOA differed among the three enthalpy categories (Kruskal–Wallis test: H = 47.56, df = 2, p < 0.001). Bonferroni-adjusted pairwise comparisons indicated higher DOA values in both the >50 to ≤58 kJ/kg group (adjusted p < 0.001) and the >58 kJ/kg group (adjusted p = 0.018) compared with the ≤50 kJ/kg group. No evidence of a difference was found between the two higher enthalpy categories (adjusted p = 1.000).

## Discussion

In the present study, a median DOA rate of 0.048 % was determined for transports of slaughter geese in Germany, with a mean of 0.161 % (SD ± 0.616 %). However, the mean should be interpreted with caution due to the non-normal distribution of the data and the presence of 18 extreme outliers (DOA > 2.0 %)**,** which accounted for only 1 % of the dataset. Analysis of the 18 extreme outliers revealed no clear or consistent patterns in ambient temperature (range: 3.1–27.8°C, mean: 18.03°C), enthalpy (range 13.88 – 58.85 kJ/kg, mean: 41.98 kJ/kg), or transport distance (range: 26.2 – 401 km, mean: 234.72 km) that could explain their elevated mortality rates based on the available data.

When compared to the limited existing literature on geese transport, the median DOA rate in this study (0.048 %) was lower than the 0.056 % reported in an earlier study (Voslářová et al., 2007a) but higher than the 0.003 % observed in a more recent study (Valkova, 2022). The latter result, however, should be treated with caution, as it was based on a relatively small sample size of 37,691 geese (Valkova, 2022). In contrast, the present study analysed 3,237,022 transported geese, providing a more robust dataset.

In comparison to recent data (2017 – 2023) on other poultry species transported in the Czech Republic, the median DOA rate for geese (0.048 %) from this study was substantially lower than the reported peak DOA rates for laying hens (1.212 %), broiler chickens (0.884 %), ducks (0.589 %), and turkeys (0.598 %) (Justova et al., 2025). Gickel et al. interpreted the comparatively low mean DOA rates in Germany for turkeys (0.14 %, SD ± 0.09) and broilers (0.09 %, SD ± 0.09) as suggesting that Germany’s transport standards exceed those observed in other countries (Gickel et al., 2024; Gickel et al., 2025). This interpretation aligns with the findings of the present study, which demonstrate comparable low mortality rates in geese during transport in Germany. An additional reason for the low DOA rates observed in this study may be that all transports arriving at the examined slaughterhouse had undergone a pre-slaughter inspection at the farm of origin. This inspection includes an assessment of transport fitness in accordance with Regulation (EU) 2019/624, conducted by official veterinarians. Geese identified as physiologically unfit for transport were excluded, thereby reducing the risk of mortality during transport. An on-farm animal inspection has the advantage that the animals can be examined more thoroughly than if they were already loaded into transport containers. In contrast, European legislation allows also live-animal inspections to be conducted only at the slaughterhouse (European Commission, 2019). This regulatory flexibility may result in animals with compromised health being transported. For such animals, transport represents a significant additional stressor, potentially exacerbating mortality risks during transit (von Holleben and von Wenzlawowicz, 2008).

Monitoring DOA rates is of high importance for assessing the current situation, establishing warning criteria, and setting progress targets (Chauvin et al., 2011). In the absence of goose-specific benchmarks, this study adopted the KTBL (KTBL, 2020d; KTBL, 2020c; KTBL, 2020b; KTBL, 2020a) and LAV (LAV, 2025) values, which were originally established for broilers, layers, and turkeys**,** for comparative purposes (Figure 1). The median DOA rate in this study was below the KTBL target value of 0.05 % (KTBL, 2020d; KTBL, 2020c; KTBL, 2020b; KTBL, 2020a), and only 4.9 % of transports recorded DOA rates above 0.55 %. According to German national law (TierSchNutzV, 2001), if the DOA rate exceeds 0.55 % for broilers, official veterinarians at the slaughterhouse must report the incident to the competent veterinary authority to investigate potential causes (LAV, 2025). However, this regulatory standard does not apply to geese and other poultry species. To uphold animal welfare principles, it should be extended to all poultry species being transported in the slaughter industry. The EFSA Panel on Animal Health and Welfare has set an even stricter threshold of 0.1 % for all domestic birds. Notably, 68.2 % of the transports in this study remained below the EFSA limit, indicating that most transports met the highest European welfare standard. However, the remaining 31.8 % exceeded the EFSA threshold, suggesting potential areas for improvement in transport practices.

In the cluster-robust regression model, transport distance was not significantly associated with DOA rate (β = −0.00018, robust SE = 0.00016, p = 0.261; Table 1), after adjustment for daily mean temperature, housing system, and year. Thus, the present data provide no evidence of an independent association between transport distance and DOA rate within the investigated range of transport distances. This finding contrasts with the earlier mentioned report from the Czech Republic on geese transports of increasing DOA with higher transport distances, which the available data could not confirm (Voslářová et al., 2007a).

The transport distances observed in the present study should also be interpreted in the context of regulatory and sector-specific requirements. Distances between the fattening farms and the slaughterhouse ranged from 9.7 to 554 km, with a median of 285.5 km (IQR 149.5–378.7 km). Assuming an average maximum driving speed of 70 km/h (LAV, 2024), this corresponds to an estimated driving time of approximately 0.1–7.9 hours, with a median of 4.1 hours (IQR 2.1–5.4 hours). These estimates refer to driving time only and do not include loading, unloading, or other potential delays.

According to Regulation (EC) No. 1/2005, poultry are treated differently from several other livestock species regarding long-distance transport. Whereas animals such as cattle must be provided with water during journeys exceeding 8 hours, poultry are not required to have access to a water supply system during transport (Council of the European Union, 2005). German legislation generally restricts the transport of animals for slaughter to a maximum of 8 hours; however, this limitation does not apply to poultry (TierSchTrV, 2009). Consequently, poultry may be transported for up to 12 hours without a water supply system, with loading and unloading times not included in this calculation (Council of the European Union, 2005; LAV, 2024). In the present study, the estimated driving times of most transports remained below 8 hours, although the longest journeys approached this duration. In organic production, Regulation (EU) 2018/848 requires transport duration to be kept as short as possible, while individual farming associations apply more restrictive recommendations (European Parliament and Council of the European Union, 2018). Two German organic associations, Bioland and Neuland, for example, recommend or require transport durations of maximal 4 hours and distances of about 200 km (Neuland, 2015; Bioland, 2024). The median distance of 285.5 km and estimated median driving time of 4.1 hours observed in the present study therefore exceeded or approached these sector-specific recommendations. These findings illustrate the challenge of reconciling animal-welfare-oriented transport recommendations with the logistical realities of goose production. Limited access to suitable regional slaughter facilities may contribute to longer transport distances, particularly in organic production, as previously discussed for livestock production more broadly (Schaack et al., 2018; Bayer et al., 2023). Improved availability of regional slaughter facilities could therefore help reduce transport distances and facilitate compliance with more stringent welfare-oriented recommendations.

The results support the view that transport duration is not the only factor that compromises animal welfare (Nielsen et al., 2011) – DOA rates are determined by a complex interplay of factors beyond mere distance, as mortality risk persists throughout the entire transport chain. Among these factors, loading conditions – such as the duration of the loading process (Cockram and Dulal, 2018) – as well as stocking density within transport containers (Nijdam et al., 2004; Chauvin et al., 2011) and the skills and careful handling by the catching personnel are known to affect mortality but were not systematically recorded in the available dataset.

Similarly, lairage times at the slaughterhouse, studied in chickens with increasing DOA due to longer lairage durations caught after midnight (Saraiva et al., 2020), and improper catching and loading as well as the truck drivers driving behaviour (KTBL, 2020d) may further contribute to stress-related losses. Due to the absence of detailed documentation on these variables, the analysed transports were categorized as routine operations, though variations in these unmeasured parameters likely influenced DOA rates over the 17-year study period.

Stocking density is particularly relevant because of its direct influence on the thermal microclimate within transport containers. At high stocking densities, poultry effectively behave as a thermal mass, generating more metabolic heat per unit surface area while reducing convective and evaporative heat dissipation, which can increase heat and moisture accumulation within transport containers (EFSA, 2022; Fahim et al., 2026). Supporting this mechanism, a study in broiler chickens reported increased transport mortality at a stocking density of 0.65 ft² per bird (9.26 % mortality) compared with chickens provided twice as much space (1.30 ft² per bird; 3.70 % mortality) (Fahim et al., 2026). However, comparable effects have not been consistently demonstrated in turkeys across the investigated stocking density range (0.001–0.010 kg/cm²) (Gickel et al., 2025), highlighting the need for species-specific studies in geese. Under German national legislation, geese weighing up to 5 kg must have a minimum floor area of 115 cm² per kilogram in the crate, with a minimum height of 25 cm. Geese weighing between 5 and 10 kg must have a minimum height of 30 cm (TierSchTrV, 2009); however, the actual stocking density of the individual transports was not available in the present dataset. Therefore, the effects attributed to thermal conditions in the present study should be interpreted with appropriate caution, as the potential influence of stocking density could not be considered.

Within this broader microclimatic context, daily mean temperature was the environmental variable that showed a significant association with DOA rate in the multivariable analysis. In the cluster-robust regression model, DOA rate increased by approximately 0.012 percentage points for each 1°C increase in daily mean temperature (β = 0.0115, robust SE = 0.0031, p < 0.001), after adjustment for transport distance, housing system, and year. The present analysis provides evidence of an overall positive association between daily mean ambient temperature and DOA rate. Although no specific temperature threshold was formally identified, the descriptive pattern suggested that the increase in DOA was most apparent at daily mean temperatures above approximately 18°C (Fig. 2). Similar effects of elevated environmental temperature have been reported in broiler chickens, in which transport and lairage mortality increased when maximum daily temperatures exceeded 17°C (Warriss et al., 2005).

Importantly, the temperature data used in the present study represented daily mean temperature recorded at a weather station approximately 10–15 km from the slaughterhouse. These data therefore provide a consistent estimate of the general environmental conditions but do not reflect the exact temperature at the time of transport, particularly during nighttime transports, nor the microclimate experienced by the geese inside the transport containers.

From an environmental perspective, enthalpy provides additional information because it integrates air temperature and humidity and therefore reflects the thermal energy content of the surrounding air. Within the concept of transport microclimate, enthalpy may consequently be more informative than temperature considered in isolation, particularly when high humidity limits the animals' ability to dissipate heat. In the present study, a separate analysis of enthalpy categories showed increasing median DOA rates with increasing enthalpy. Median DOA was 0.047 % at ≤50 kJ/kg, 0.13 % at 50 to <58 kJ/kg, and 0.28 % at >58 kJ/kg. The highest enthalpy category contained only 12 transports, and these findings should therefore be interpreted cautiously. Furthermore, enthalpy was not included simultaneously with daily mean temperature in the multivariable regression model because of the close relationship between these environmental variables. Nevertheless, the pattern supports further investigation of enthalpy as a potential indicator of adverse thermal conditions during goose transport and is consistent with evidence that thermal stress in poultry depends on the combined effects of temperature, humidity, and ventilation (Oladokun and Adewole, 2022).

The thermal microclimate inside poultry transport containers is determined by the temperature, humidity of the incoming air, airflow velocity, and the heat and moisture produced by the animals (Mitchell and Kettlewell, 2009). Temperature, humidity and CO₂ levels can vary significantly depending on the position of the transport container on the vehicle. Transport crates in the middle are subject to higher stress, because the animals rely solely on the airflow from the vehicle for thermoregulation (Holmes, 2020). During the journey, air flows in at the rear of the vehicle and moves over the animals towards the front, where it exits. As soon as the vehicle comes to a standstill, ventilation stops and heat and moisture dissipation can only occur via natural convection (Mitchell and Kettlewell, 2009). Most vehicles are fitted with windbreak tarpaulins. These are closed in cold weather and provide protection against, for example, splashing water. However, they minimise air inflow and outflow and can lead to an uneven distribution of thermal conditions within the vehicle (Cockram and Dulal, 2018; EFSA, 2022).

Direct measurements of temperature and humidity within transport containers would therefore provide a substantially more accurate assessment of the thermal exposure experienced by the geese than external weather data alone. Such measurements would allow crate-level variation in heat and moisture accumulation, ventilation, and enthalpy to be evaluated throughout transport. Although continuous monitoring under commercial transport conditions may be challenging, it should be considered in future studies. Monitoring enthalpy has previously been proposed as a means of identifying potentially critical thermal conditions, particularly at high outdoor temperatures (Holmes 2020). However, based on the present data, specific enthalpy thresholds for goose transport cannot yet be established. Future studies combining continuous crate-level measurements with information on stocking density, transport duration, loading conditions, and DOA would be required to determine whether enthalpy can serve as a reliable operational indicator of heat-stress risk.

In the current study, no significant differences in DOA rates were observed between geese sourced from conventional versus organic farms. Housing system was not significantly associated with DOA rate in the cluster-robust regression model after adjustment for daily mean temperature, transport distance, and year. Organic geese accounted for approximately 3.8 % of the sample, a proportion that underrepresents organic production systems in Germany, where organic production accounted for 14.86 % of goose farming in 2023 (Statistisches Bundesamt, 2026b). Accordingly, the absence of a significant association should be interpreted cautiously because of the pronounced imbalance between conventional and organic transports in the present dataset. Despite stricter welfare standards in organic farming – such as extended rearing periods of 20 weeks for organic geese (Naturland, 2024) and enhanced housing conditions – the health status of poultry from organic systems did not differ meaningfully from conventional systems (Bayer and von Meyer-Höfer, 2021).

For instance, Neuland mandates an investigation into transport losses exceeding 0.35 % (Neuland, 2015), yet the median DOA rates for organic geese (0.042 %) and for conventional geese (0.049 %) remain well below this threshold. Organic standards, such as greater space allowances, lower flock stocking densities (max. 2500 geese per barn, 21 kg/m² live weight), and mandatory outdoor access (15 m² per goose) (Naturland, 2024) were not associated with a detectable reduction in transport mortality in the present dataset. However, for the transports examined, information was available only on whether a farm was organic, not on the organic farming association to which it belonged.

The absence of a difference between conventional and organic geese in this study supports findings of a former study (Bayer and von Meyer-Höfer, 2021), which identifies transport and slaughter phases as critical weak points in organic poultry systems. Despite higher welfare standards during rearing, organic production often fails to differentiate itself from conventional production during these logistical stages. Exposure to extreme temperatures, traffic delays, and inadequate pre-transport health assessments undermining the potential advantages of organic husbandry (Bayer and von Meyer-Höfer, 2021) and likely contribute to this convergence in DOA rates.

Year was likewise not significantly associated with DOA rate in the multivariable regression model, providing no evidence of a systematic linear temporal trend in DOA rates over the 17-year study period after adjustment for daily mean temperature, transport distance, and housing system. This finding suggests that the observed association between daily mean temperature and DOA rate was not attributable to a general long-term increase or decrease in DOA over the study period.

In summary, several limitations should be considered when interpreting the present findings. Daily mean temperature and humidity were derived from a weather station near the slaughterhouse and therefore represent general environmental conditions rather than the actual microclimate within the transport containers. Cluster-robust standard errors accounted for repeated transports from the same fattening farm, although this approach does not estimate farm-specific effects. Future studies should include direct crate-level measurements of temperature and humidity to better characterize thermal exposure during transport.

This study provides valuable insights into a large-scale dataset of transports of slaughter geese in Germany, representing one of the most comprehensive analyses of its kind with over 3.2 million transported animals. While not all critical variables identified in the literature, such as loading procedures, stocking density or lairage times could be systematically evaluated due to data limitations, daily mean temperature was significantly associated with DOA rates, whereas transport distance, housing system, and year were not. Additional analyses indicated differences in DOA rates among enthalpy categories. Repeated transports from the same fattening farm were accounted for through cluster-robust statistical inference. However, the enthalpy findings should be regarded as complementary to the multivariable temperature analysis rather than as evidence of an independent enthalpy effect. Overall, these findings emphasize the importance of thermal conditions during the transport of geese to slaughter and highlight the need for future studies incorporating direct measurements of the microclimate experienced by the animals.

## Author Contribution

Benedikt Sauer: Contributed to the conceptualization and design of the experimental setup and methodology, data validation, data curation, statistical data analysis, data visualization, and writing of the initial manuscript draft. He also incorporated the contributions and comments from the co-authors and reviewers into the final manuscript. He approved the final version of the manuscript and accepts accountability for all aspects of the work. Isabelle Ruhnke: Contributed to validation of the study and reviewing of the manuscript. She approved the final version of the manuscript and accepts accountability for all aspects of the work. Katharina Charlotte Jensen: Contributed to the formal analysis, visualization of the results and was involved in drafting the manuscript.She approved the final version of the manuscript and accepts accountability for all aspects of the work. Kenan Hizal: Contributed to the formal analysis, visualization of the results and was involved in drafting the manuscript. He approved the final version of the manuscript and accepts accountability for all aspects of the work. Eva-Maria Saliu: Contributed to the methodology and validation of the study, and reviewing of the manuscript, and supervision. She approved the final version of the manuscript and accepts accountability for all aspects of the work. All authors have made substantial intellectual contributions to the work, have approved the final version of the manuscript, and agree to be personally and publicly accountable for all aspects of the work, including ensuring that questions related to the accuracy or integrity of the work are appropriately investigated and resolved.

## Declaration of AI use

During the preparation of this work the author(s) used DeepL and **Mistral AI’s Le Chat** to improve readability and language. After using these tools/services, the author(s) reviewed and edited the content as needed and take(s) full responsibility for the content of the publication.

## Disclosures

The authors declare that they have no known competing financial interests or personal relationships that could have appeared to influence the work reported in this paper.
